# Neuroleukemiosis: Two Case Reports

**DOI:** 10.7759/cureus.1529

**Published:** 2017-07-31

**Authors:** Vlad Voin, Shehzad Khalid, Sebastian Shrager, R. Shane Tubbs, Robert Greiner, Krishnamoorthy Thamburaj, Elias Rizk

**Affiliations:** 1 Research Fellow, Seattle Science Foundation; 2 Department of Anatomical Sciences, St. George's University School of Medicine, Grenada, West Indies; 3 Department of Anatomical Research, St. George's University School of Medicine, Grenada, West Indies; 4 Neurosurgery, Seattle Science Foundation; 5 Hematology-Oncology, Penn State Milton S. Hershey Medical Center; 6 Department of Neuroradiology, Penn State Milton S. Hershey Medical Center; 7 Department of Neurosurgery, Penn State Milton S. Hershey Medical Center

**Keywords:** neuroleukemiosis, acute myeloid leukemia, peripheral nerve, brachial plexus, neuropathy

## Abstract

Extramedullary tumors composed of myeloblasts or monoblasts can present in various locations. Patients with a history of acute myeloid leukemia (AML) can present with neuropathic pain and no evidence of relapse of their leukemia. Neuroleukemiosis is a form of extramedullary tumor present in the peripheral nervous systems (PNS) of leukemia patients. We report two AML patients who were in remission and later presented with neurological symptoms due to neuroleukemiosis with negative bone marrow biopsies.

## Introduction

Acute myeloid leukemia (AML) is a malignant disease of hematopoietic stem and progenitor cells [1]. It is characterized by the accumulation of immature blasts instead of functional myeloid cells in the bone marrow and peripheral blood [1]. The annual incidence ranges from 3 to 8 persons per 100,000, with a median age of onset of 67 years [1]. Extramedullary tumors composed of myeloblasts or monoblasts have been referred to as chloromas and occur in around 5% of AML cases [2-3]. When they involve the peripheral nervous system (PNS), the condition is called neuroleukemiosis [3-6].

The literature contains only a handful of case reports of isolated leukemic masses infiltrating the PNS. Such masses usually appear after patients are in remission and there are no other signs of relapse [6]. We report two cases of patients diagnosed with AML who later presented with neurological symptoms due to neuroleukemiosis. We also review the pertinent diagnostic tools and treatment options for patients who present with these findings.

## Case presentation

Case report 1

**Figure 1:**
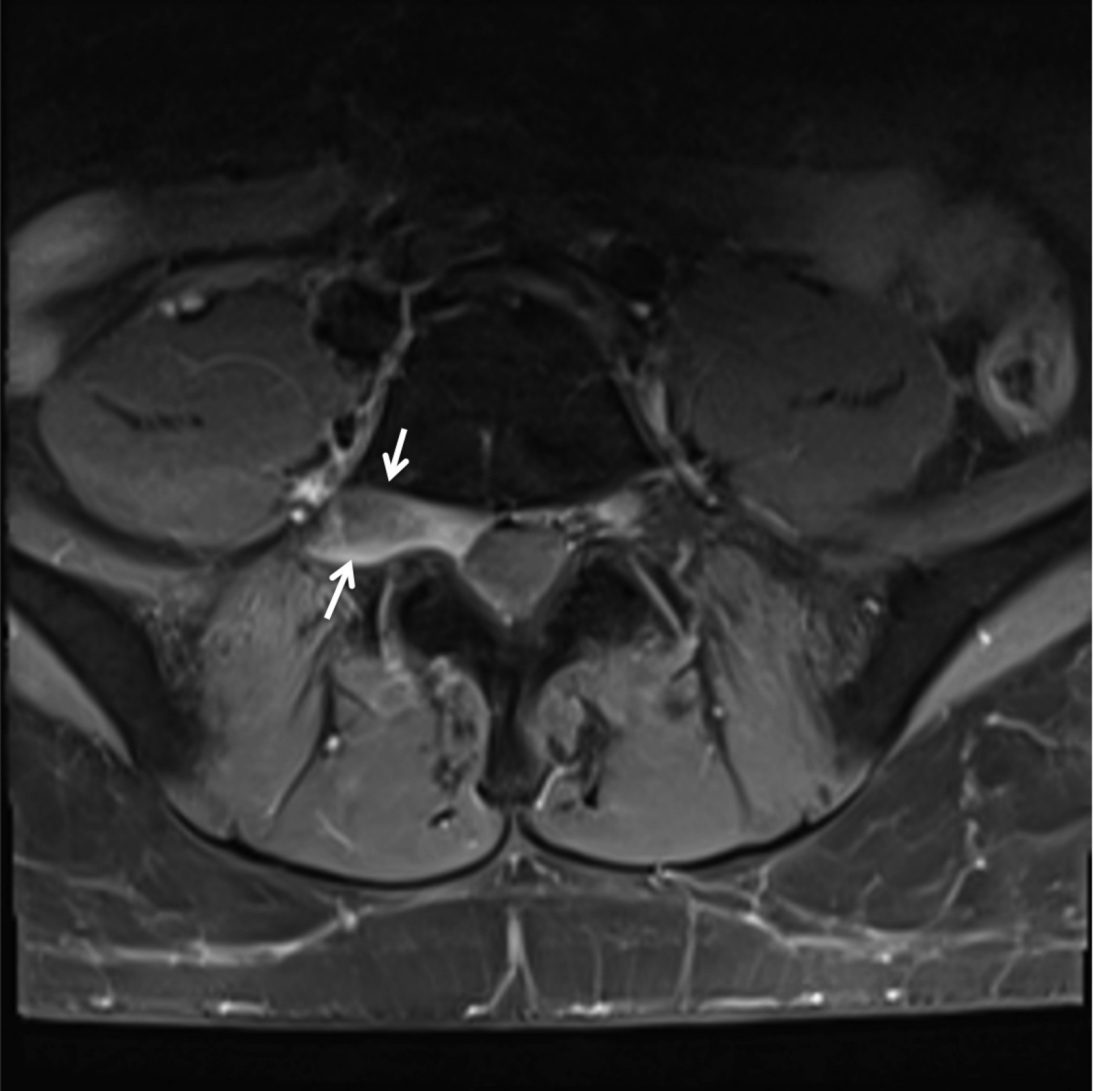
Axial magnetic resonance imaging (MRI) of patient 1. Representative axial MRI of patient 1 noting expansile lesion of right lumbar nerve root.

An 18-year-old male with a history of AML diagnosed in 2010, status post bone marrow transplant and donor lymphocyte infusion (DLI), presented with a one-year history of progressive deep left shoulder and left upper arm pain extending into the C8 dermatome. Epidural injection did not relieve his pain and in a follow-up exam, he had contralateral symptoms and right S1 radicular symptoms. Analysis of the cerebrospinal fluid (CSF) was positive for leukemic cells. Initial magnetic resonance imaging (MRI) showed only C7 nerve involvement on the left. Repeat MRI showed multiple expansive soft tissue masses extending through the neural foramina involving the right C4-C5 and C5-C6, the left neural foramina at C6-T3 into the paraspinal tissues, and the right L5-S1 neural foramina (Figure 1). Biopsy of the paraspinal tissue was consistent with chloroma. The patient underwent systemic chemotherapy and craniospinal radiation. The three-month follow-up MRI showed complete resolution of the masses except for residual nerve enlargement and enhancement at C6-C7.

Case report 2

**Figure 2:**
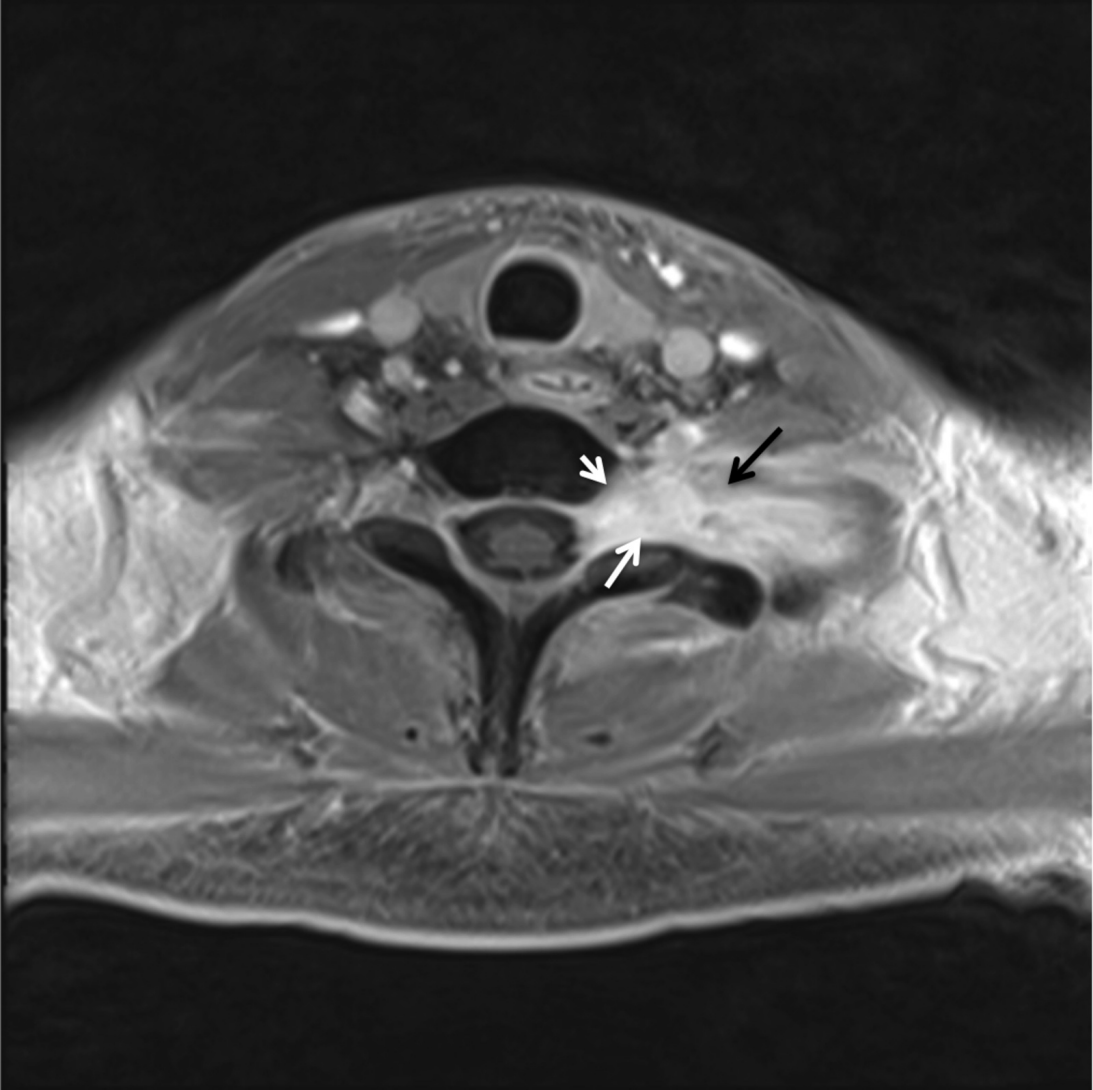
Axial magnetic resonance imaging (MRI) of patient 2. Representative axial MRI of patient 2 noting lesion of left cervical nerve root and adjacent soft tissues.

A 16-year-old male with a history of AML, status post stem cell transplant in 2014, presented with left upper extremity radiculopathy that started in November 2016. This progressed to the weakness of the left limb with severe uncontrolled pain and numbness. His MRI showed an infiltrative lesion with necrotic areas involving the left brachial plexus and left neck and posterior spinal muscles, extending from C2 to T1 and into the spinal epidural space via the left C5-C6 (Figure 2) to T1-T2 neural foramina. There was a similar signal-limited lesion in the neural foramen on the right side at C5-C6 and C6-C7 with likely involvement of the right brachial plexus. Biopsy of left neck mass revealed myeloid sarcoma and blast cells. The patient was started on azacitidine and sorafenib. While his neuropathic pain improved, his prognosis was poor due to the relapse of the AML.

## Discussion

The most common locations of extramedullary tumors in patients with AML are the skin, soft tissue, bone, and lymph nodes. Central nervous system (CNS) and PNS involvement is rare [3-7]. Both the PNS and CNS have barriers that make the spread of leukemic cells to those sites unlikely.

The spread of leukemia to the peripheral nervous system is still debated. A blood–nerve barrier (BNB), similar to the blood–brain barrier (BBB), separates the circulating blood from the peripheral nerves [6-7]. The BNB is comprised of endoneurial vascular endothelial cells, a basement membrane and is surrounded by pericytes [6]. The tight intercellular junctions make it difficult for leukemic cells to penetrate. However, malignant cells that do penetrate the BNB would be protected from systemic chemotherapeutic drugs that cannot penetrate it and therefore have no access to the PNS [3,7-8].

Wang, et al. suggested that in patients with isolated neuroleukemiosis, a hematogenous spread of blast cells into the PNS is more likely than longitudinal dissemination from nerve root [6]. They proposed that pathogenic cells can penetrate an impaired BNB, and leukocytes can enter the PNS irrespective of any disturbance in the BNB [6,9]. Once the leukemic cells make it into the PNS they are in an ideal location to thrive, making them very difficult to treat.

Non-resolving neuropathic symptoms of pain, numbness, and weakness can be the first signs of neuroleukemiosis. In the cases reported here, both the patients presented with some type of neuropathic symptoms. In the first case, the patient presented with progressive shoulder and arm pain over a year. The symptoms of left arm weakness, however, seemed to progress much more rapidly in the second case than the first. With only neuropathic symptoms present, it is easy to misdiagnose a leukemic mass infiltrating the PNS, especially if the blood smear and bone marrow biopsy are negative for leukemia, which was not the case with these patients.

Differential diagnosis for neuroleukemiosis includes Guillain-Barre syndrome (GBS), neurotoxicity from chemotherapy, and abscess [3,6]. Nerve conduction studies can help differentiate axonal damage from demyelination, but the most helpful tests are radiological exams, specifically MRI and positron emission tomography (PET) [6]. MRI and PET scans allow all relevant parts of the PNS and surrounding structures to be examined, which is helpful for locating the lesions and assessing the effect of treatment [10]. The lesions have a typical radiographic appearance on MRI and PET, with T1 isointensity, T2 hyperintensity, enhancement after gadolinium, and strong fluorodeoxyglucose (FDG) uptake [4]. High-resolution ultrasound is a new method providing better spatial resolution of the PNS than MRI [10]. Nerve biopsy is the gold standard and a full pathological assessment and comparison to the original bone marrow pathology should be done [3,6].

Treatment decisions depend on the type of leukemia, patient symptoms, and extent of metastasis. Local infiltration and local tumors are most frequently treated with local radiation therapy and surgical decompression to alleviate symptoms [7,10]. Most treatments combine systemic chemotherapy with intrathecal chemotherapy and radiation therapy for long-term results [4]. This treatment allows for penetration of the CSF as well as targeting mass lesions.

## Conclusions

In patients with unexplained new-onset neuropathy and a history of AML, neuroleukemiosis must be included in the differential diagnosis. Even when there is apparent bone marrow remission and blood smears are negative, the possibility of CNS or PNS involvement by relapsing leukemia cannot be ruled out. Because it is difficult for systemic chemotherapeutic drugs to penetrate the BNB, the PNS makes an ideal location for blast cells to infiltrate and thrive after the patient has been in remission for many years.
